# A Case of Thoracic Epidural Angiolipoma Causing Severe Spinal Cord Compression With Neurologic Manifestations

**DOI:** 10.7759/cureus.45305

**Published:** 2023-09-15

**Authors:** Kali A King, Tiffany Tsao, Ahmed Abdulrahim, Alec Hildenbrand

**Affiliations:** 1 Internal Medicine, Creighton University School of Medicine, Omaha, USA; 2 Pathology and Laboratory Medicine, Creighton University School of Medicine, Omaha, USA

**Keywords:** spinal cord compression, thoracic tumor, angiolipoma, spinal tumor, spinal epidural angiolipoma

## Abstract

A spinal epidural angiolipoma is a rare, benign tumor of adipocytes and blood vessels that accounts only for a small percentage of all spinal axis tumors. We report a case of a 44-year-old male who presented with three months of progressive decreased sensation and strength from about six cm above the umbilicus down to his feet bilaterally. He presented to the emergency room when he could no longer walk. He also had neurogenic urinary retention and likely neurogenic constipation. Physical exam was notable for decreased sensation, decreased strength, and increased patellar reflexes bilaterally. MRI of the thoracic spine showed a posterior epidural mass that spanned from T2 to T3, measuring 1.2 x 1.7 x 4.3 cm, and severely compressed the spinal cord posteriorly. The patient underwent an urgent laminectomy for decompression and mass resection. Pathology was consistent with an angiolipoma. Postoperatively, he experienced a drastic improvement in strength and gross motor skills. The sensation had a partial return following surgery and continued to improve over the hospital stay. In general, the literature reports significant symptomatic improvement in patients with spinal epidural angiolipomas after surgical resection.

## Introduction

A spinal epidural angiolipoma is a benign tumor made up of mature adipocytes and thin-walled blood vessels. It is a variant of lipomas with an extreme degree of vascular proliferation. Angiolipomas occur mostly in subcutaneous tissue and rarely in the spinal epidural region. They account for only 0.04-1.2% of all spinal axis tumors and about 2-3% of all extradural spinal tumors [1]. There are two types of spinal epidural angiolipomas - infiltrating and non-infiltrating. The infiltrating type invades into the surrounding soft tissue and is often mistaken for an aggressive tumor [2]. The non-infiltrating type, as seen in the present case, does not invade the surrounding tissue. These masses are well-visualized on MRI imaging. Patients will often undergo laminectomy and total surgical resection to return to normal function [1].

## Case presentation

Our patient is a 44-year-old male with a past medical history significant for morbid obesity (BMI of 55) and tobacco use of 10 pack-years. The patient initially presented to the emergency department with three months of recurrent falls, lower extremity weakness, and numbness that had worsened in the last two weeks. His symptoms began with numbness, which he first noticed upon waking up one morning. He described his numbness as starting at a horizontal line about six inches above his umbilicus and extending down to his feet bilaterally. This included perineal and penile numbness. Then, gradually over several weeks, he started to have lower extremity weakness and began using a cane. When he presented, he could no longer walk, and he could barely sit up in bed without assistance. He had difficulty relaxing his sphincter tone during defecation, which manifested as severe constipation. He also had worsening difficulty initiating urination. Of note, he denied any back pain and had no point tenderness on examination. He had not had any recent illness, trauma, or surgery on his back.

After being admitted, his bladder scan showed a post-void residual urine volume of 1,000 ml, and a Foley catheter was inserted. On examination, he was pleasant and was not in distress over his condition. His neurological exam was significant for 2+ strength in bilateral lower extremities, most prominently in the hip girdle with abduction. There was a significant decrease in fine touch, pain, and vibration sensations bilaterally from the torso down, with worsened sensation distally. He had hyperreflexia with 3+ patellar reflex bilaterally. His cranial nerves were intact. Strength and sensation were normal in the bilateral upper extremities.

**Figure 1:**
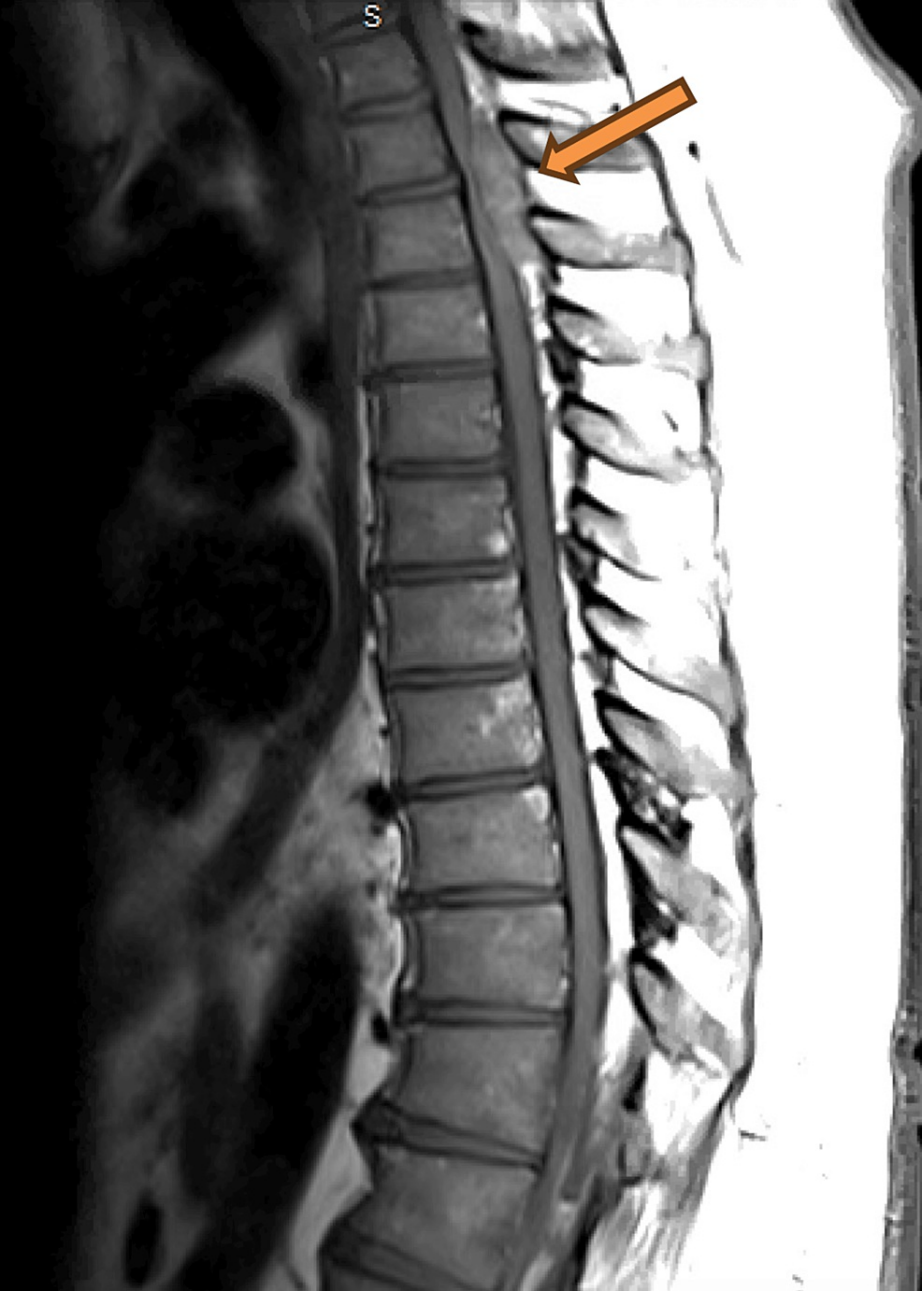
T1-weighted image of MRI thoracic spine with enhancing posterior epidural mass spanning from T1-2 to T3-4.

**Figure 2:**
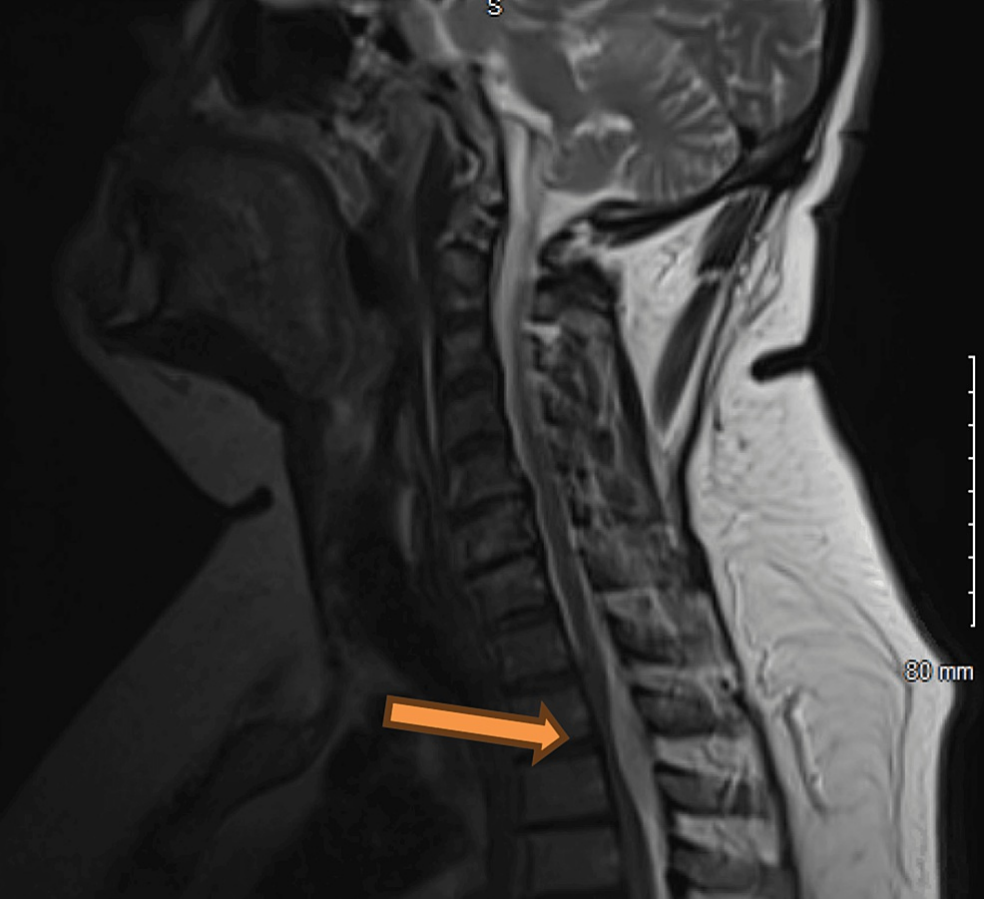
T2-weighted image showing the mass.

An MRI of the thoracic and lumbar spine showed a homogeneously enhancing mass within the posterior epidural space, spanning from the superior endplate of T2 to the inferior plate of T3 (Figures 1-2). This mass measured up to 1.2 x 1.7 x 4.3 cm in maximum dimension, severely compressing the spinal cord at these levels. Additionally, there was a narrowing of the remainder of the thoracic and lumbar spinal canal secondary to prominent epidural fat. A CT scan of the abdomen and pelvis was done for initial concern of neoplasm, which yielded no significant findings.

**Figure 3:**
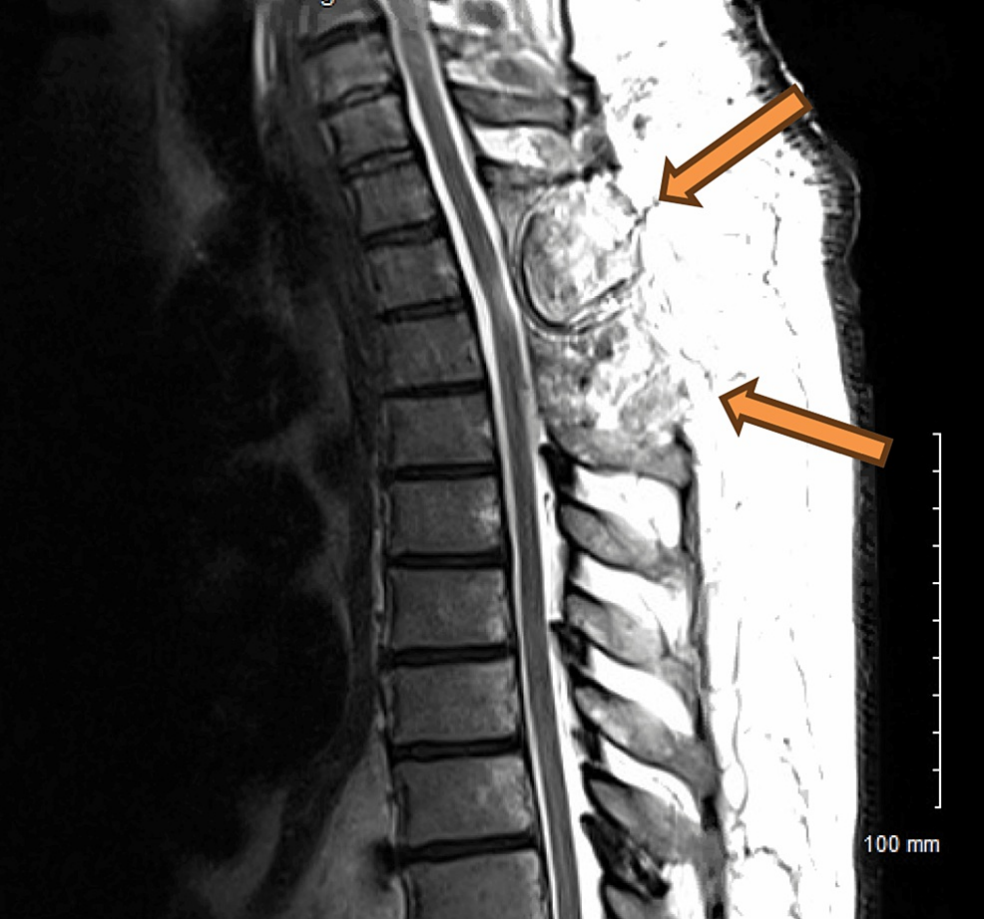
T2 weighted image showing interval T2 and T3 laminectomy and resection of dorsal epidural mass decompressing the spinal. Changes of myelomalacia at the T2-3 level.

**Figure 4:**
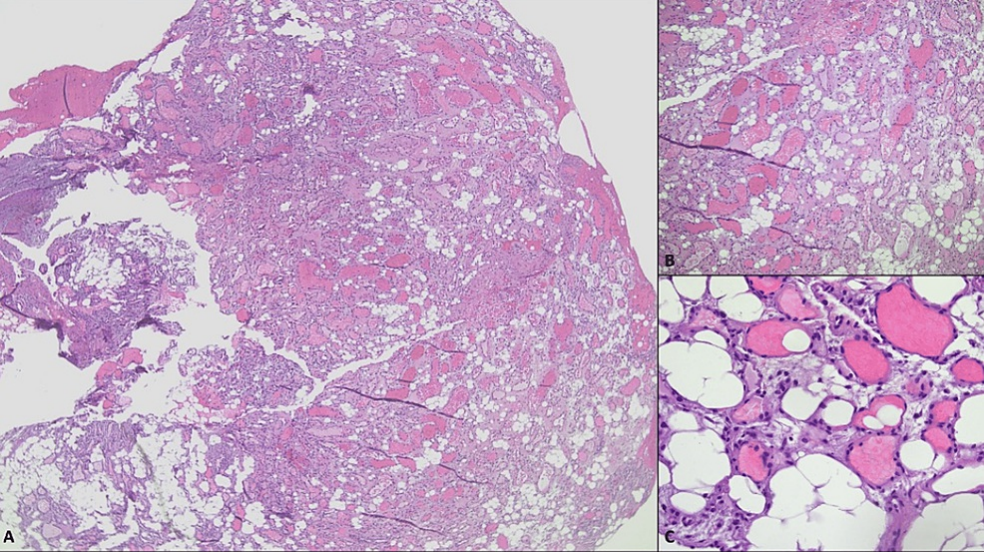
A, B, and C. Clusters of thin-walled blood vessels filled with intraluminal fibrin thrombi and surrounded by mature adipose tissue. 4x, 10x, and 40x magnifications, respectively.

On hospital day two, the neurosurgery team took the patient for a decompressive laminectomy with mass resection and biopsy. Upon visualization during the surgery, the mass appeared largely hemorrhagic with aspects of fat. Intraoperative frozen section revealed a lipomatous/angiomatous mass without gross signs of malignancy. Post-operatively, there was immediate improvement in neurological symptoms seen on examination. Immediately after surgery, the patient endorsed a subjective 30% improved sensation from the torso down, most improved proximally. A follow-up MRI thoracic spine completed 48 hours after surgery showed findings consistent with T2-T3 laminectomy and complete removal of the epidural mass. It also showed volume loss of the dorsal left side of the spinal cord at T2, which indicated myelomalacia (Figure 3). The pathology report described the mass as fibroadipose tissue with prominent blood vessels, with the diagnosis of angiolipoma (Figure 4).

Following the surgery, the patient experienced drastic symptom improvement for the rest of his hospital stay. While working with physical therapy, he was able to transition from sitting on the edge of the bed to taking steps with a walker, walking across the room with a walker, and sitting in a chair. In his final physical therapy note before leaving for rehabilitation, his ODI score was calculated to be 10 points or 20%, meaning minimal disability. His severe neurogenic urinary retention resolved. His post-void residual urinary volume was within normal limits after the removal of the Foley catheter three days after the operation. His constipation, which possibly had a neurogenic component, resolved with stool softeners. He was discharged to an acute rehab facility to continue physical therapy on hospital day nine.

## Discussion

Angiolipomas are very rarely found in the spinal canal. The cause of these tumors is not well understood. Proposed mechanisms suggest they may be derived from local pluripotent stem cells that have divergent differentiation caused by an unknown stimulus [3]. Other proposed causes include congenital malformations or disorganized growth of local tissue [3]. The most common presenting symptoms are numbness and weakness, and they often escalate to paraparesis. A literature review found the peak incidence of angiolipomas to be around 46 years of age and a time of diagnosis from symptom onset ranging from one day to 17 years [3]. MRI imaging is the best method for detection given its anatomic soft tissue contrast and visualization of spinal cord compression [4]. Pathology is needed to confirm the type of tumor and rule out possible malignancy. Total surgical resection is the treatment of choice to relieve symptoms. In the most recent systematic review of 60 patients, 85% had full recovery of the motor deficits and symptoms manifested in the preoperative period, 10% of patients showed mild improvement, and 5% of patients presented symptoms unchanged [1]. Outcomes can be incredibly favorable, as seen in this case.

## Conclusions

This case report highlights the rarity and clinical significance of spinal epidural angiolipomas. These benign tumors, though uncommon, can lead to significant neurological deficits if not promptly diagnosed and managed. Through a multidisciplinary approach involving neurosurgery and radiology, our patient underwent successful surgical resection, resulting in a remarkable improvement in neurological function and quality of life. This case brings forward the importance of considering epidural angiolipomas in the differential diagnosis of spinal cord compression and the favorable outcomes achievable with timely intervention. Further research and awareness are warranted to better understand this condition and optimize its management.
